# Long noncoding RNA CASC9.5 promotes the proliferation and metastasis of lung adenocarcinoma

**DOI:** 10.1038/s41598-017-18280-3

**Published:** 2018-01-08

**Authors:** Jianlong Zhou, Haiping Xiao, Xinglong Yang, Hao Tian, Zhiyong Xu, Yu Zhong, Limin Ma, Weimin Zhang, Guibin Qiao, Jing Liang

**Affiliations:** 1 Department of the Sixth Medicine, Guangzhou General Hospital of Guangzhou Military Command, Guangdong Province, China; 2 Cancer Research Institute, Southern Medical University, Guangdong Province, China; 3 Department of Oncology, Guangzhou General Hospital of Guangzhou Military Command, Guangdong Province, China; 4 Department of Thoracic surgery, Guangzhou General Hospital of Guangzhou Military Command, Guangdong Province, China; 5 Department of TCM, Guangzhou General Hospital of Guangzhou Military Command, Guangdong Province, China

## Abstract

Long non-coding RNAs (lncRNAs) are important regulatory factors in tumor development and progression. The lncRNA CASC9.5 is located on chromosome 8 and has a total length of 1316 bp. CASC9.5 plays a tumor-promoting role in the development and progression of brain tumor and colon cancer; however, **limited research has** been conducted on the role of this lncRNA in lung adenocarcinoma. The present study analyzed 44 lung adenocarcinoma specimens and 2 lung cancer cell lines. It was found that CASC9.5 expression levels were significantly higher in lung cancer tissues and cells compared with normal lung tissues. In addition, the expression level of CASC9.5 was closely related to the TNM (**tumor**, **node and metastasis)** stage of lung adenocarcinomas, tumor size, tumor metastasis and tumor metabolism. Moreover, results of the *in vivo* and *in vitro* experiments all demonstrated that CASC9.5 promoted lung adenocarcinoma cell proliferation and metabolism by regulating the expression levels of cyclin D1, E-cadherin, N-cadherin and β-catenin. In summary, the present study demonstrated that high levels of CASC9.5 expression promote the proliferation, metastasis and metabolism of lung adenocarcinoma cells and might serve as a prognostic indicator. The present study provides novel findings regarding the diagnosis and treatment of lung adenocarcinoma.

## Introduction

At present, lung cancer is the most common primary malignancy of the lung. The vast majority of lung cancers originate from the epithelium of the bronchial mucosa. Therefore, lung cancer is also known as bronchogenic carcinoma. In terms of incidence, lung cancer ranks first among all human malignancies, and smokers experience a high incidence of lung cancer. Over the past 50 years, the incidence and mortality of lung cancer have increased rapidly in all countries around the world, especially in industrially advanced countries. Currently, lung adenocarcinoma is the most common type of lung cancer^1,2^. The 5-year survival rate of lung cancer is approximately 16.6%. Lung cancer ranks first as the cause of cancer deaths in males. By 2025, it is estimated that the number of Chinese patients with lung cancer will reach 1 million, and China may become first in lung cancer incidence worldwide. The metabolic status of the cells in lung adenocarcinoma is of great significance to cancer progression and diagnosis. Understanding the molecular mechanisms and pathways of cancer cell metabolism is conducive to enhancing the efficacy of lung adenocarcinoma treatments and improving the prognosis of lung adenocarcinoma. The invasion, metastasis and metabolism of cancer cells in their microenvironment are related in a complex way and are regulated by a variety of factors. The most important mechanism responsible for regulating the metabolism and metastasis of cancer cells is the epithelial-mesenchymal transition (EMT). The role of the EMT in cancer is similar to what occurs in embryonic development. In cancer, EMT reduces the connection between cells, enhances their migratory capability and induces a stromal cell phenotype in epithelium-derived cancer cells. In addition, the EMT is accompanied by changes in a number of important markers, such as decreased E-cadherin expression and increased N-cadherin expression. Importantly, decreased E-cadherin expression is a critical step in the progression of well-differentiated adenomas toward an aggressive phenotype^3^. To date, most studies have attempted to study the regulation of the EMT in cancer cells by investigating special proteins and pathways, such as epidermal growth factor (EGF), transforming growth factor beta (TGF-β) and fibroblast growth factor (FGF)^4^. However, recent studies indicate that long non-coding RNAs (lncRNAs) may be related to the pathogenesis of non-small cell lung cancer (NSCLC)^5,6^.

These studies have provided a new idea for the investigation of the molecular mechanisms underlying the metastasis and metabolism of lung adenocarcinoma. With the emergence of gene chips and the rapid development of high-throughput sequencing technologies, it has been found that more than 90% of the mammalian genome can be transcribed into non-coding RNAs. Among these RNAs, a class with lengths of more than 200 nt and an extremely low probability of encoding proteins are known as lncRNAs^7^. Although the available studies on lncRNAs are far from exhaustive, it is evident that many lncRNAs play important roles in cellular processes and in the regulation of apoptosis and cell metabolism^8–10^. Studies have shown that abnormal regulation of lncRNAs is related to the development and metabolism of a variety of cancers, including lung cancer^11–13^. Such studies provide a comprehensive understanding of the important biological role of the EMT in the development and metabolism of lung adenocarcinoma. However the specific mechanism of each lncRNA is not clear.

The lncRNA CASC9.5 is located on chromosome 8 and has a length of 1316 bp. Recently, second-generation sequencing analysis showed that CASC9.5 functions as a noncoding proto-oncogene and is involved in the occurrence of lung adenocarcinoma^11^. Our study found that the expression level of CASC9.5 was significantly higher in lung adenocarcinoma tissues in comparison to the paracancerous tissues. It was also found that CASC9.5 expressions levels are related to tumor TNM (tumor, node and metastasis) stage tumor size and lymph node metastasis. In addition, CASC9.5 might regulate cell growth and metastasis by regulating cyclin D1, E-cadherin, N-cadherin and β-catenin. Moreover, our study demonstrated that CASC9.5 is capable of binding to DNA methyltransferase 1 (DNMT1), providing a reasonable explanation for CASC9.5-mediated regulation of E-cadherin expression. Our results indicate that increased expression of CASC9.5 in lung adenocarcinoma may play an important role in the growth and metastasis of cancer cells.

Our results suggested that high levels of CASC9.5 expression promote the proliferation, metastasis and metabolism of lung adenocarcinoma cells, CASC9.5 would serve as a prognostic indicator due to its important role in lung adenocarcinoma.

## Results

### CASC9.5 is highly expressed in lung adenocarcinoma tissues and is correlated with poor prognosis

**Figure 1:**
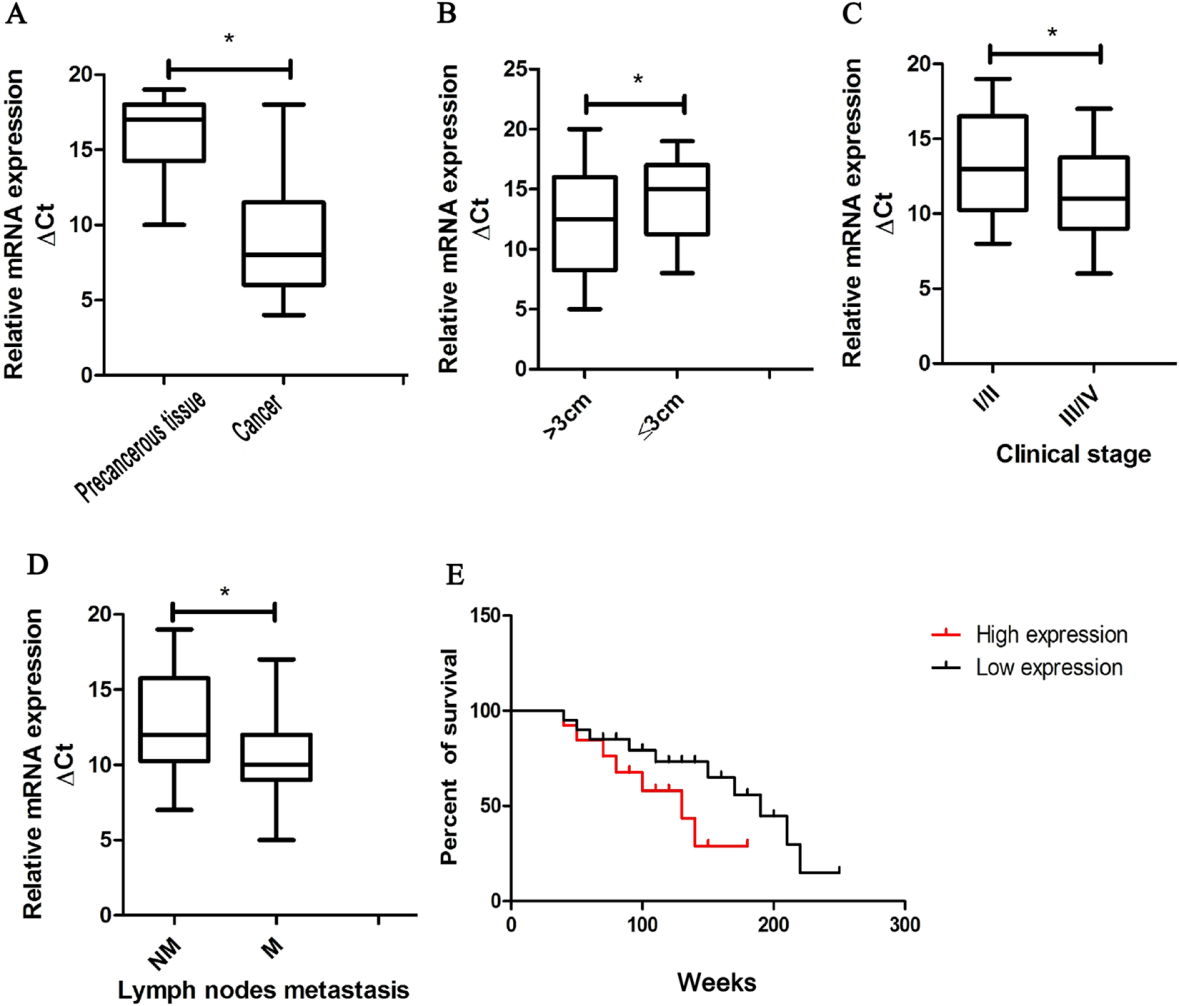
(**A**) Examination of the relative expression level of long non-coding RNA (lncRNA) CASC9.5 in 40 lung adenocarcinoma specimens and paracancerous tissue samples using fluorescence-based quantitative polymerase chain reaction (qRT-PCR). (**B**–**D)** lncRNA CASC9.5 was highly expressed in lung adenocarcinomas that had reached a large volume, were in advanced clinical stages or had undergone lymph node metastasis. (**E**) Survival analysis demonstrated that patients with high expression of lncRNA CASC9.5 suffered a poorer prognosis. *p < 0.05, **p < 0.01.

To determine whether difference exist in the expression levels of CASC9.5 between lung adenocarcinoma tissues and paracancerous tissues, we collected 40 lung adenocarcinoma tissue specimens. We examined CASC9.5 expression in cancer tissues and paracancerous tissues at the RNA level using fluorescence-based quantitative polymerase chain reaction (qRT-PCR). The results showed that the expression level of CASC9.5 was significantly increased in cancer tissues (P = 0.02; Fig. 1A). In addition, we assessed the role of CASC9.5 in the clinical diagnosis, treatment and prognosis of lung adenocarcinoma. It was found that CASC9.5 expression levels were closely related to the size of lung adenocarcinoma, the TNM stage and lymph node metastasis (Fig. 1B–D). In contrast, CASC9.5 expression was not correlated with tumor differentiation, patient gender or patient age. Survival analysis of the 40 cases of lung adenocarcinoma revealed that patients whose tumors expressed high levels of CASC 9.5 had poorer prognoses compared to patients with tumors expressing low levels of CASC 9.5 (Fig. 1E). The above results indicate that CASC9.5 may play an important role in the development and progression of lung adenocarcinoma.

### Expression status of CASC9.5 in non-small cell lung cancer (NSCLC) cells

**Figure 2:**
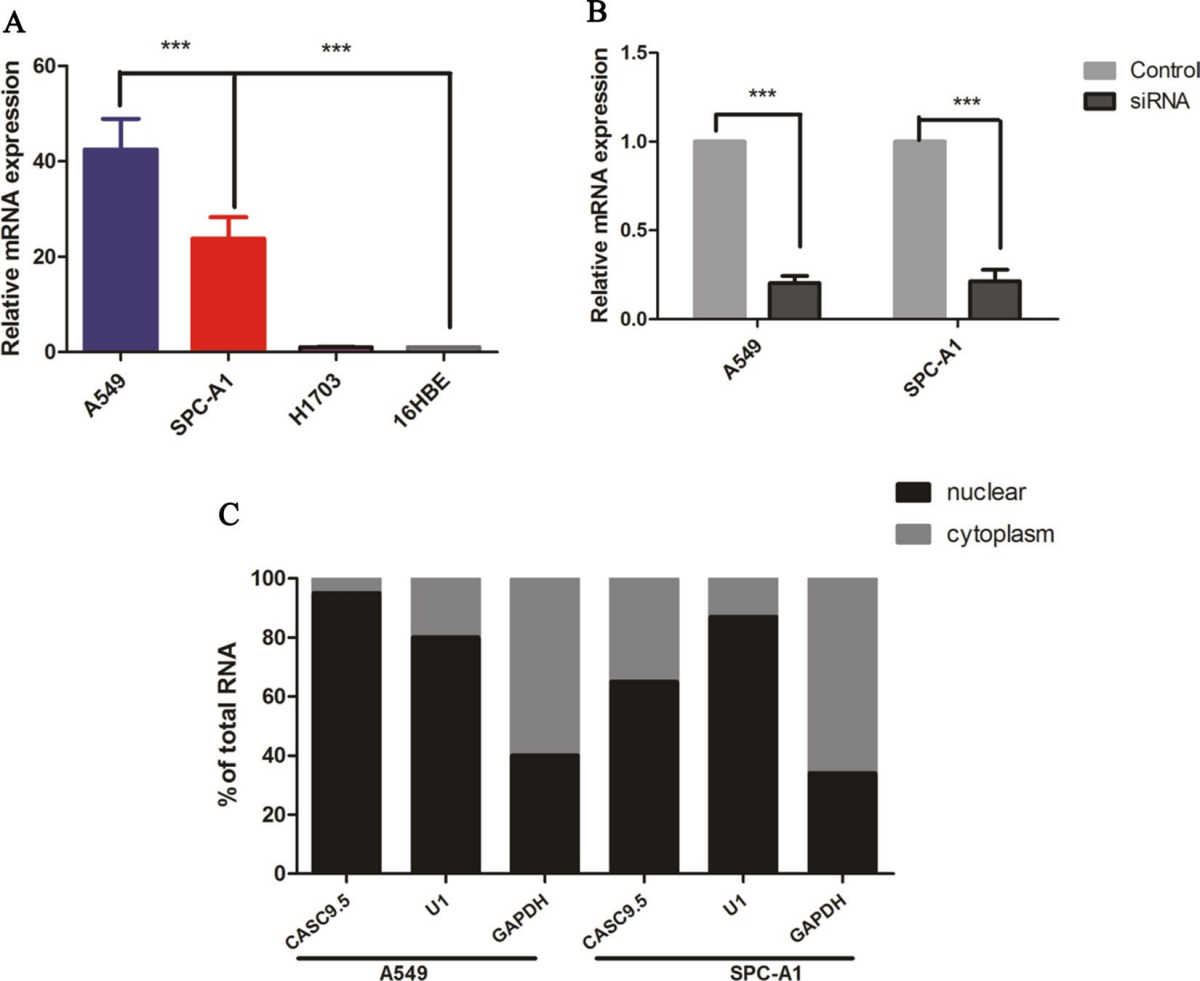
(**A**) Examination of the expression level of long non-coding RNA (lncRNA) CASC9.5 in non-small cell lung cancer (NSCLC) cell lines using fluorescence-based quantitative polymerase chain reaction (qRT-PCR). 16HBE cells are normal bronchial epithelial cells. (**B**) The knockdown (KD) of the expression of lncRNA CASC9.5 in A549 and SPC-A1 cells using RNA interference (RNAi). (**C**) qRT-PCR analysis revealed that lncRNA CASC9.5 was primarily located in the nucleus. Glyceraldehyde 3-phosphate dehydrogenase (GAPDH) was used as a cytoplasmic marker, while U1 RNA was used as a nuclear marker. *p < 0.05, **p < 0.01, ***p < 0.001.

To investigate the role of CASC9.5 in the progression of lung cancer, qRT-PCR was performed to examine the expression level of CASC9.5 RNA in 4 NSCLC lines. It was found that CASC9.5 expression levels were significantly higher in the 2 lung adenocarcinoma cell lines A549 and SPC-A1 in comparison to other lung cancer cell lines (Fig. 2A). Small interfering RNAs (siRNA) were employed to downregulate the expression of CASC9.5 in A549 and SPC-A1 lung adenocarcinoma cells. Forty-eight hours after transfection with siRNA, CASC9.5 expression levels in A549 and SPC-A1 cells decreased by more than 70% compared to the control group (Fig. 2B). qRT-PCR was then performed to determine the cellular location of CASC9.5 in A549 and SPC-A1 cells. In this experiment, glyceraldehyde 3-phosphate dehydrogenase (GAPDH) was used as a cytoplasmic marker, while U1 RNA was used as a nuclear marker. The results showed that the expression level of CASC9.5 was higher in the nucleus than in the cytoplasm (Fig. 2C). The findings indicate that CASC9.5 is primarily localized in the nucleus and may be involved in transcriptional regulation.

### Effect of CASC9.5 on the proliferation of lung adenocarcinoma cells

**Figure 3:**
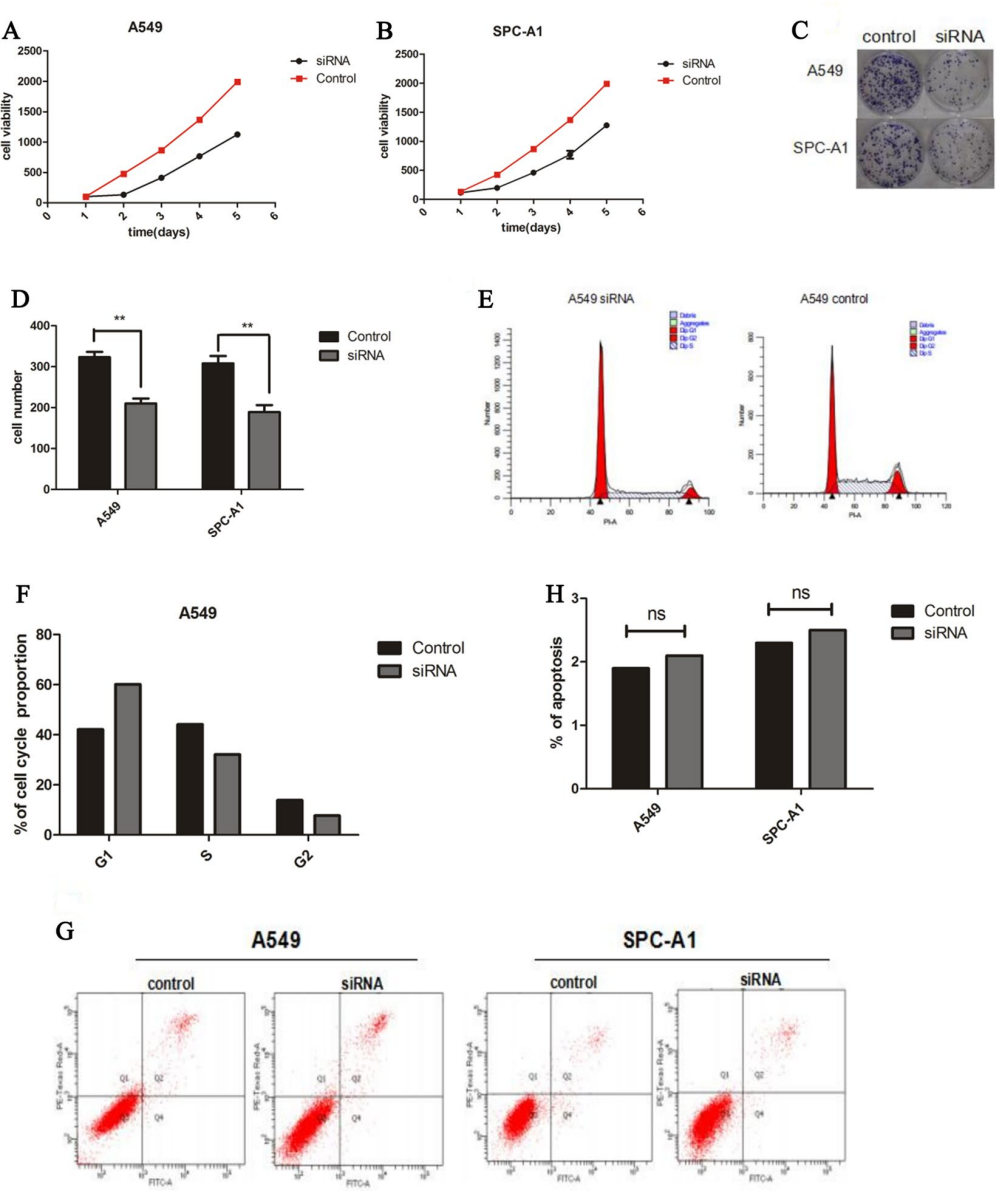
(**A**–**D**) A cell proliferation assay (CCK-8) and plate clone formation assay demonstrated that CASC9.5 knockdown (KD) inhibited the proliferation of lung adenocarcinoma cell lines. (**E**–**F**) Cell cycle analysis showed that CASC9.5 KD increased the percentage of G1-phase cells and reduced the percentage of S-phase cells. (**G**–**H**) An apoptosis assay showed that CASC9.5 KD had no effect on apoptosis. *p < 0.05, **p < 0.01.

With the rapid development of second-generation sequencing techniques and various types of chip analyses, the biological functions of lncRNAs have been increasingly explored. We conducted a variety of experiments on CASC9.5-knockdown (KD) lung adenocarcinoma cell lines to examine the effect of this lncRNA on lung adenocarcinoma. The results of a Cell Counting Kit-8 (CCK-8) assay showed that compared to the control group, the KD of CASC9.5 in the lung adenocarcinoma cell lines A549 and SPC-A1 resulted in reduced cell viability and proliferative capability (Fig. 3A,B). A plate clone formation assay also showed that cell lines expressing low levels of CASC9.5 were less clonogenic than the control group (Fig. 3C,D). Subsequently, the cell lines were subjected to cell cycle analysis. It was found that the percentage of G1-phase cells was increased while the percentage of S-phase cells was decreased in A549 CASC9.5-KD cells compared to the control group. However, a similar phenomenon was not observed in SPC-A1 cells (Fig. 3E,F). In addition, apoptosis was examined using flow cytometric analysis. No significant difference was detected between the CASC9.5-KD group and the control group (Fig. 3G,H). Therefore, it can be concluded that CASC9.5 regulates the proliferation of lung adenocarcinoma cells by regulating the G1 phase of cell cycle.

### *In vivo* experiments show that CASC9.5 knockdown (KD) inhibits the growth of lung adenocarcinoma

**Figure 4:**
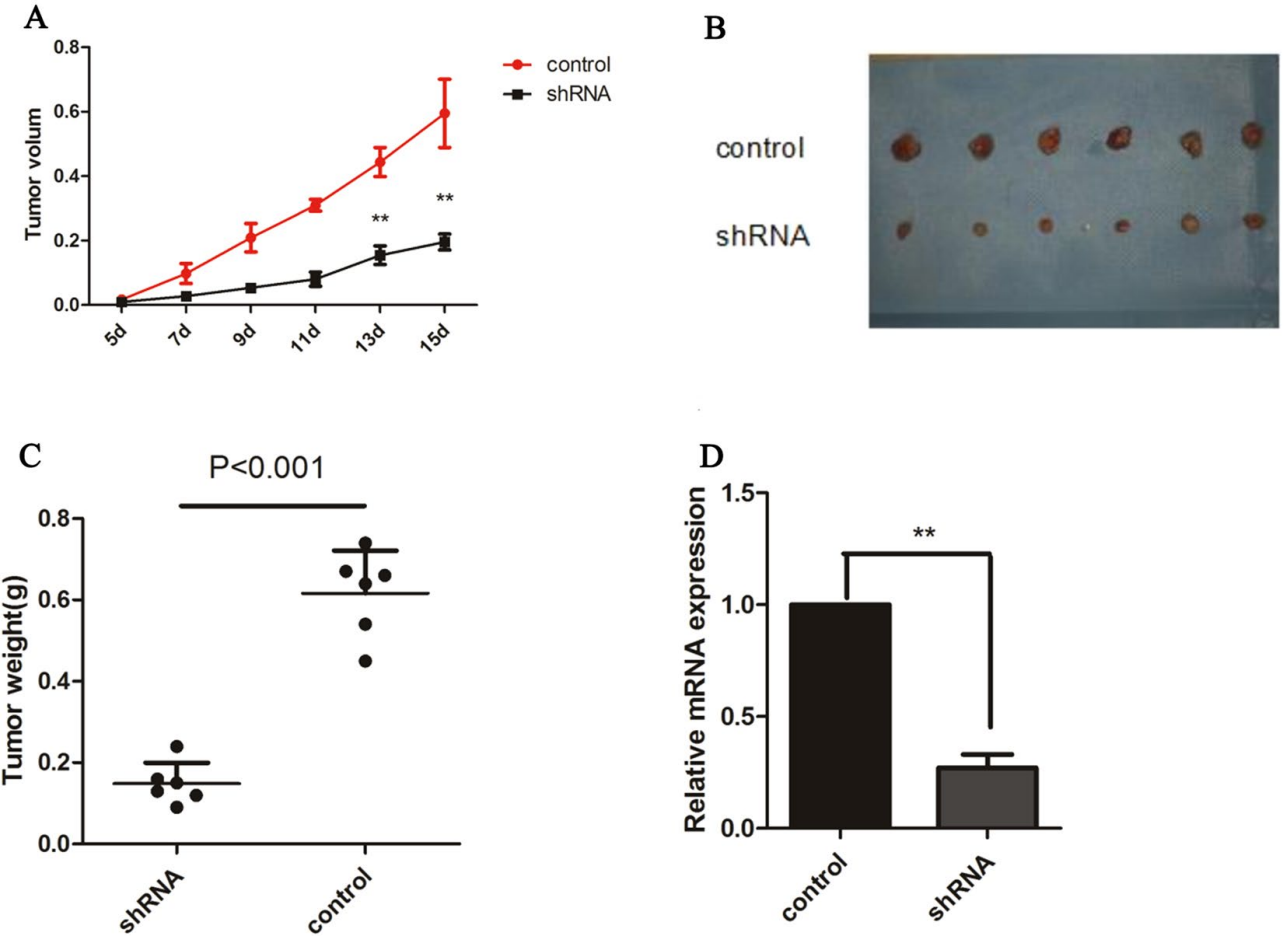
(**A**–**C**) Examination of subcutaneous tumor formation in nude mice revealed that CASC9.5 knockdown (KD) inhibited the proliferation of lung adenocarcinoma. (**A**) Comparison of the volume of the xenograft tumors. (**B**) Comparison of the xenograft tumors. (**C**) Comparison of the weight of the xenograft tumors. (**D**) Comparison of CASC9.5 expression level in xenograft tumors. *p < 0.05, **p < 0.01.

The *in vitro* experiments described above demonstrated that high levels of CASC9.5 expression in lung adenocarcinoma cells promoted cancer cell growth and progression. To verify whether such effects also manifest *in vivo*, a small hairpin RNA (shRNA) technique was employed to construct stable A549 cell lines expressing low levels of CASC9.5. The control group was established by transfecting cells with non-targeting control shRNA (Fig. 4A). The stable cell lines were then subcutaneously inoculated into nude mice to assess the *in vivo* capacity of the cells to form tumors. At 15 d after inoculation, a series of tumor parameters were compared. Compared to the control group, the volume and the weight of the xenograft tumors were both reduced in the group of mice that were inoculated with cells expressing low levels of CASC9.5 (Fig. 4B–D). The *in vivo* experiments demonstrate that the downregulation of CASC9.5 expression inhibits the proliferation of lung adenocarcinoma, which is consistent with the results of the *in vitro* experiments.

### Both *in vivo* and *in vitro* experiments demonstrate that CASC9.5 knockdown (KD) inhibits the metastasis of lung adenocarcinoma

**Figure 5:**
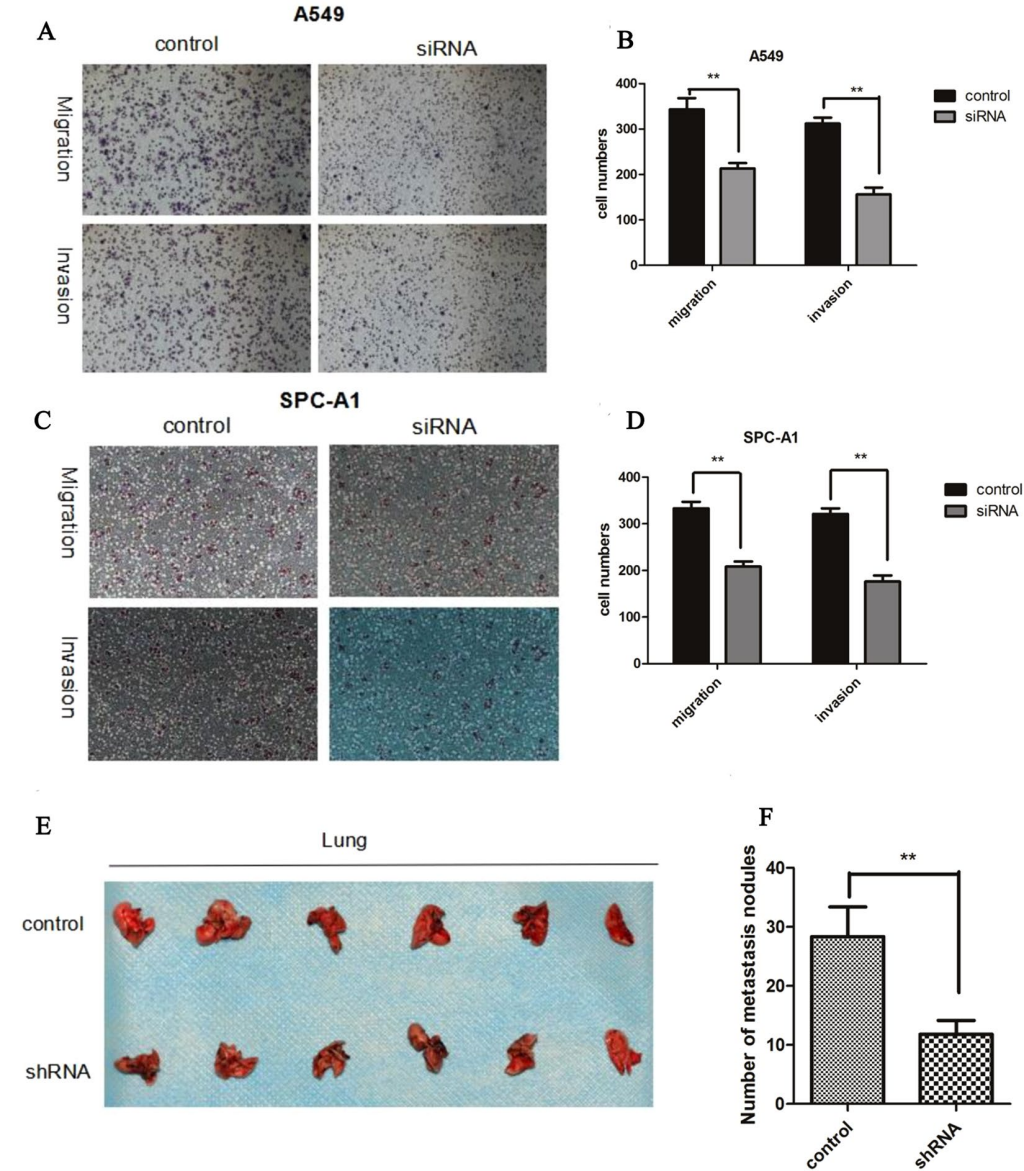
(**A**–**D**) Transwell migration and invasion assays demonstrated that CASC9.5 knockdown (KD) in the lung adenocarcinoma cell lines A549 and SPC-A1 inhibited the migratory and invasive capabilities of the tumor cells. (**E**–**F)** *In vivo* experiments showed that KD of the expression of CASC9.5 in lung adenocarcinoma cell lines significantly inhibited the metastasis of lung adenocarcinoma. *p < 0.05, **p < 0.01.

To determine whether inhibition of CASC9.5 expression promotes the metastasis and invasion of NSCLC, a Matrigel invasion assay and Transwell migration assay were conducted. Compared with the control group, A549 cells in which CASC9.5 expression was decreased using siRNA exhibited reduced migratory and invasive capabilities (Fig. 5A,B). Similar results were obtained using the SPC-A1 cell line (Fig. 5C,D). The above findings demonstrate that CASC9.5 plays an important role in the metastasis and invasion of lung adenocarcinoma. To investigate the effect of CASC9.5 on the metastasis of lung adenocarcinoma cells *in vivo*, nude mice were given tail vein injections of stable A549 cells in which CASC9.5 expression was knocked down with shRNA. At 8 w after injection, the number of lymph node metastases on the surface of the lung was counted. Compared with the control group, the CASC9.5 KD group had a significantly lower number of lymph node metastases (Fig. 5E,F). The results of the *in vivo* experiments further confirmed the functions of CASC9.5 identified *in vitro*.

### DNMT1 binds to CASC9.5

**Figure 6:**
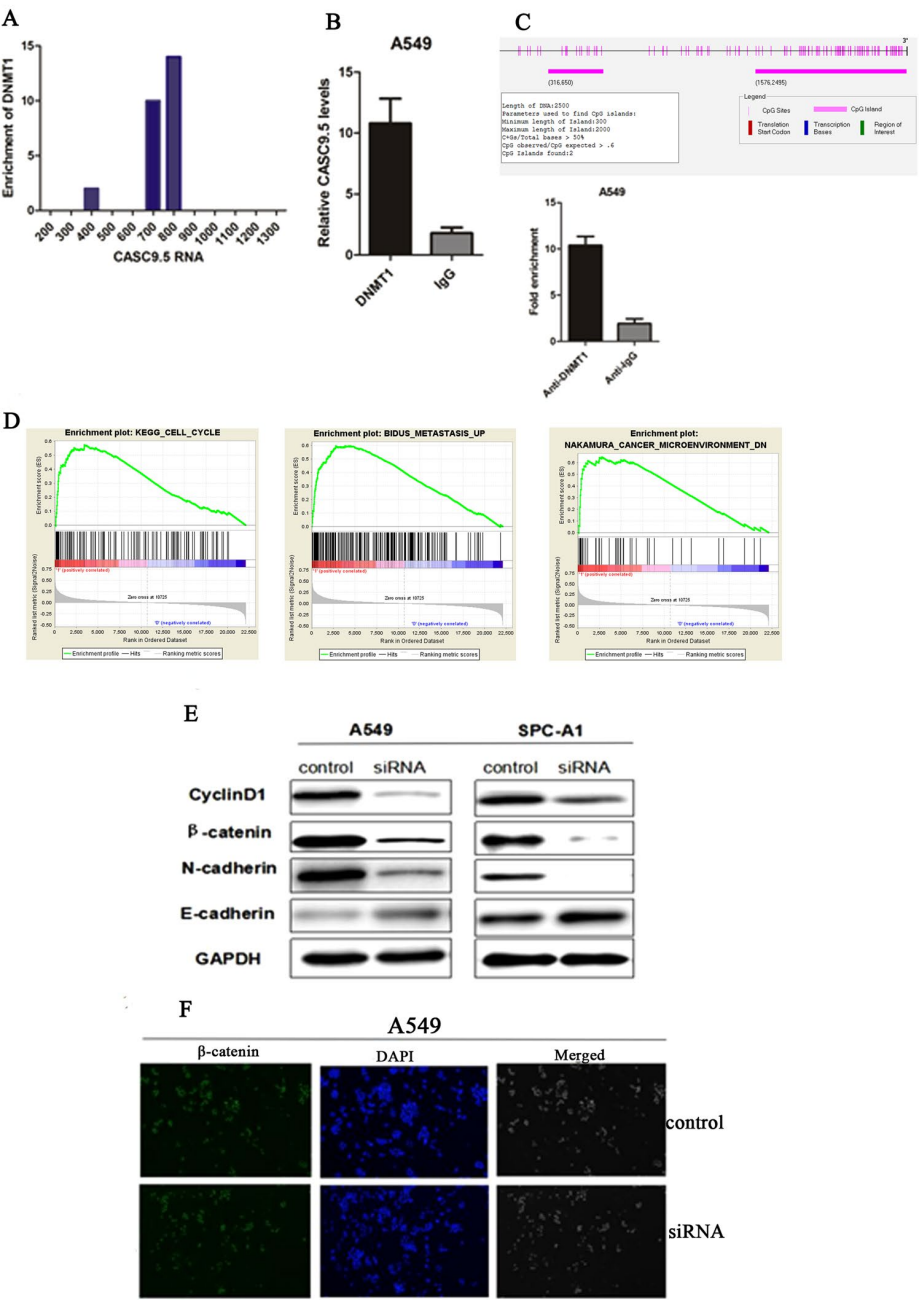
(**A**) Bioinformatic analysis revealed an enrichment in DNMT1 sites on CASC9.5. (**B**) RNA immunoprecipitation assay showed that DNMT1 interacts with CASC9.5. (**C**) Bioinformatic analysis revealed that the CpG islands were present in the promoter region of E-cadherin. (**D**) Gene set enrichment analysis (GSEA) showed that CASC9.5 expression was related to tumor metastasis, tumor microenvironment and cyclin genes. (**E**) Western blot analysis of the expression status of cyclin D1, E-cadherin, N-cadherin and β-catenin in CASC9.5-knockdown (KD) A549 and SPC-A1 lung adenocarcinoma cell lines. (**F**) Immunofluorescence analysis of the expression of β-catenin in A549 lung adenocarcinoma cells after siRNA treatment. *p < 0.05, **p < 0.01.

To explore the mechanisms by which CASC9.5 regulates the proliferation and invasion of lung adenocarcinoma cells, bioinformatics techniques were employed for the identification of proteins that are potentially capable of binding to the full-length CASC9.5. The results showed that CASC9.5 was enriched with DNMT1 binding sequences (Fig. 6A).

DNA methylation is an epigenetic modification that plays a key role in transcriptional regulation and is associated with most human malignancies^14,15^ DNMT1 is an indispensable factor in DNA methylation and plays an essential role in the maintenance of epigenetic inheritance and in epigenetic replacement^16^. DNMT1 also plays an important role in tumor progression^17,18^. To investigate any possible connection between DNMT1 and CASC9.5, RNA immunoprecipitation was performed in A549 cells using an antibody specific for DNMT1. It was found that CASC9.5 was enriched in the DNMT1-RNA precipitates (Fig. 6B). The result demonstrated an interaction between DNMT1 and CASC9.5.

### CASC9.5 affects the EMT of lung adenocarcinoma cells

The experiments described above revealed that CASC9.5 regulates the proliferation of lung adenocarcinoma cells primarily by regulating the cell cycle. Therefore, western blotting was performed to examine the expression level of cyclin D1. Compared to the reference group, the expression level of cyclin D1 was significantly decreased in the cells with siRNA-mediated CASC9.5 KD. The results indicate that CASC9.5 may regulate cell cycle progression through cyclin D1. The EMT is one of the most important events in epithelial tumor metastasis and invasion^19^. E-cadherin is an important factor in intercellular adhesion and exerts an inhibitory effect on tumor metastasis. UCSC bioinformatic analysis revealed that CpG islands are enriched in the promoter region of E-cadherin and our results showed that DNMT1 could directly bind to E-cadherin promoter region (Fig. 6C). Gene set enrichment analysis (GSEA) showed that CASC9.5 expression was related to tumor metastasis, tumor microenvironment and cyclin genes (Fig. 6D).Therefore, western blotting was performed to examine the expression level of E-cadherin and 2 other EMT markers (N-cadherin and β-catenin) in CASC9.5-KD A549 cells. CASC9.5 KD enhanced the expression of E-cadherin and reduced the expression of N-cadherin and β-catenin (Fig. 6E). In addition, immunofluorescence analysis also showed that CASC9.5 KD in A549 cells reduced the expression level of β-catenin (Fig. 6F). Therefore, it is likely that E-cadherin is an important downstream regulatory factor of CASC9.5 and is regulated by DNMT1.

## Discussion

An increasing number of studies have explored the important regulatory effects of lncRNAs on mammalian genes. lncRNA dysfunction may be involved in epigenetic alterations and contribute to tumor formation and metastasis. Therefore, the study of the tumor-related lncRNAs may provide novel ideas for the diagnosis and treatment of tumors.

The lncRNA CASC9.5 is located on chromosome 8 and has a length of 1316 bp. Recently, second-generation sequencing analysis shows that CASC9.5 functions as a noncoding proto-oncogene and is involved in the occurrence of lung adenocarcinoma^20^. A large amount of lncRNA were found in the tumor and all the results were named by MiTranscriptome compendium, which is available for scientific use on a public web site, www.mitranscriptome.org. In this site, search results showed a significant positive association between CASC9.5 and lung adenocarcinoma. The present study discovered for the first time that CASC9.5 expression is related to lung adenocarcinoma. Specifically, the expression level of CASC9.5 was observed to be significantly higher in lung adenocarcinoma tissue in comparison to paracancerous tissues. More importantly, CASC9.5 expression levels were drastically elevated in cancers that became large in size and exhibited a high degree of malignancy. The above findings suggest that CASC9.5 may serve as a diagnostic factor for lung cancer and plays a role in the diagnosis of lung cancer. In addition, CASC9.5 KD in lung adenocarcinoma cell lines significantly inhibited cell viability, the cell cycle, as well as migration and invasiveness. In summary, the present study indicates that CASC9.5 may function as a cancer-promoting factor and play an important role in the development and progression of lung adenocarcinoma. Many other lncRNAs have also been proven to play a role in cancer progression, and such lncRNAs are being used as cancer markers. For example, it has been shown that increased expression of metastasis-associated lung adenocarcinoma transcript 1 (MALAT1) is correlated with the progression and metastasis of lung cancer^5^. Another notable example is the Hox transcript antisense RNA (HOTAIR), which is generally highly expressed in most human tumors^21^. Therefore, effective blocking of the production of these lncRNAs may be an efficient strategy for the treatment of cancer. Our *in vivo* animal experiments further confirmed that CASC9.5 effectively inhibited the proliferation and metastasis of lung adenocarcinoma.

Although CASC9.5 is considered to be a cancer-promoting factor, the mechanisms of action of CASC9.5 in tumor progression remain unclear. To explore the molecular mechanism by which CASC9.5 promotes the metastasis and proliferation of lung adenocarcinoma, the present study investigated potential targets related to cancer proliferation and metastasis. The results of the present study confirmed that cyclin D1 was a functional target of CASC9.5 in lung adenocarcinoma. Cyclin D1 is one of the most important proteins in cell cycle regulation and plays a critical role in the progression of a series of human cancers (including lung cancer)^22,23^. After binding to cyclin-dependent kinase 4 (CDK4) and CDK-interacting protein/kinase inhibitor protein (CIP/KIP), cyclin D1 enters into the nucleus and phosphorylates the retinoblastoma tumor suppressor protein (pRb). In this way, cyclin D1 promotes cell cycle progression from the G1 phase into the S phase^24^. The present study found that cyclin D1 is a downstream regulatory factor of CASC9.5 and plays a role in regulating the growth of lung adenocarcinomas. To further explore the function of CASC9.5, additional studies should build a more complete map of the expression and cellular functions of lncRNAs, thereby developing a better understanding of various tumor properties.

Previous studies have shown that enhancing the expression of lncRNA CASC9.5 affects the migratory and invasive capabilities of brain tumor cells. However, the underlying molecular mechanisms of these effects remain unclear. To explore how CASC9.5 enhances the metastasis and invasiveness of lung adenocarcinoma cells, the present study examined the potential target proteins related to cell migration and invasion. The EMT plays an important role in promoting the metastasis and invasion of epithelial tumors, including lung adenocarcinomas^11,12,25,26^. The EMT is primarily characterized by the reduced expression of E-cadherin and increased expression of N-cadherin, leading to an enhanced metastatic and invasive capability of tumor cells^27,28^. The findings of present study indicated that the metastasis and invasion of tumor cells were strongly associated with EMT-related markers. The present findings also confirm that the downregulation of CASC9.5 expression inhibits the metastasis and invasion of NSCLC through the EMT, which may indicate a therapeutic target for anti-metastatic treatments.

The functions of lncRNAs often depend on their binding proteins. For example, it has been reported that the lncRNA HOXA transcript at the distal tip (HOTTIP) directly targets the WD repeat-containing protein 5 (WDR5) and the WDR5/mixed lineage leukemia (MLL) protein complex, thereby inducing gene transcription and histone H3 lysine 4 (H3K4) trimethylation^29^. Furthermore, HOTAIR and X-inactive specific transcript (Xist) have been reported to interact with the chromosome remodeling complex PRC2 (polycomb repressive complex 2), thereby inhibiting gene expression^14^. The present study found that CASC9.5 was primarily located in the nucleus and mainly bound to DNMT1, the most important DNA methyltransferase in mammalian cells. The main functions of DNMT1 in human tumor cells are maintaining gene methylation status and silencing gene expression^15,16^. Di Ruscio *et al*. found that DNMT1 may be blocked from methylating specific genes by interacting with RNAs^18^.

In summary, the present study found that CASC9.5 is a cancer-promoting factor and stimulates the progression of lung adenocarcinoma via the EMT. Therefore, CASC9.5 may serve as a diagnostic biomarker of lung adenocarcinoma and be a potential treatment target for this type of tumor. *In vitro* and *in vivo* experiments showed that CASC9.5 KD in lung adenocarcinoma effectively inhibited tumor growth and metastasis. The results demonstrate the possibility that CASC9.5 may be a target for the treatment of lung adenocarcinoma.

## Materials and Methods

### Collection and treatment of specimens

lung adenocarcinoma specimens were collected from 44 patients with lung adenocarcinoma treated at the Department of Thoracic surgery, Guangzhou General Hospital of Guangzhou Military Command of PLA between 2010 and 2014. Of these specimens,four were excluded from this study because of incomplete patient medical records. The patients ranged in age from 45 to 70. The specimens were evaluated by two or more qualified pathologists in a double-blind manner, and adjacent noncancerous tissues were used as normal controls. The specimens were stored at −80 °C before use. This study was approved by the Ethics Committee of Guangzhou General Hospital of Guangzhou Military Command and Each patient signed the written informed consent before enrollment in the study. The procedures involving animals and their care were conducted in conformity with NIH guidelines (NIH Pub. No. 85–23, revised 1996). All the methods were carried out in accordance with the relevant guidelines and regulations about animals and humans.

### Cell culture and transfection

Two lung adenocarcinomar cell lines, A549 and SPC-A1, were purchased from American Type Culture Collection (ATCC). Both cell lines were cultured in complete 1640 medium (Gibco) which contained 100 U/ml penicillin, 10% fetal bovine serum (FBS; Gibco), and 100 mg/ml streptomycin (Gibco). For transfection, Lipofectamine 2000 (Invitrogen) was used according to the package insert.

### RNA extraction and fluorescence-based quantitative polymerase chain reaction

TRIzol reagent were used to extract RNA according to Product Manual (ambion).

Reverse transcriptase-PCR (RT-PCR) was performed according to the user manual (Takana, SYBRII), and the primer sequences were as follows: glyceraldehyde-3-phosphate dehydrogenase (GAPDH): forward primer 5′-GGGAGCCAAAAGGGTCAT-3′′, reverse primer 5′-GAGTCCTTCCACGATACCAA-3′.

### Western blotting

Radioimmunoprecipitation assay (RIPA) lysis buffer (Fude Biological Technology Co., Ltd) was used to extract protein as follows: cells in one well of a 6-well plate were digested with 0.25% trypsin (Gibco) and centrifuged at 1000 rpm (Thermo); then the supernatant was discarded, and the pellet was washed once with phosphate-buffered saline (PBS) (Gibco) at room temperature. After centrifugation, the supernatant was discarded, 120 μl of RIPA lysis buffer was added, and the cells were lysed on ice for 30 minutes, followed by centrifugation at 4 °C and 12 000 rpm for 15 minutes. Next, 100 μl of the supernatant was transferred to a new 1.5 ml Eppendorf (EP) tube (Corning), and the remaining supernatant was used to determine the protein concentration via a bicinchoninic acid assay (BCA assay; Beyotime Biotechnology). For western blot assays, 50 μl of loading buffer containing dithiothreitol (DTT) was added into a new EP tube; the protein samples were denatured at 100 °C in a metal bath for 10 minutes and then loaded on to the gels. The primary E-cadherin, N-cadherin, cyclin D1and β-catenin antibody were a mouse-anti-human antibody (Abcam) diluted to 1/1 000 in 5% bovine serum albumin (BSA); the secondary antibody was a goat anti-mouse antibody (Abcam) diluted to 1/10 000 in Tris-buffered saline-Tween 20 (TBST). GAPDH (37 kDa) was used as an internal control. All antibodies and internal controls were used according to the manufacturer’s instructions.

### Immunofluorescence analysis

The cells were fixed with 4% paraformaldehyde. Rabbit anti-β-catenin antibody (1:100 dilution; Cell Signaling Technology, Inc.) was used as the primary antibody, and fluorescein isothiocyanate (FITC)-labeled anti-rabbit IgG (1:200 dilution; Abcam Company) was used as the secondary antibody. The images were taken using a fluorescence microscope (Nikon).

### Cell proliferation assay

The CCK-8 assay was conducted using 96-well plates (Corning Inc.). Briefly, the cells were seeded into the 96-well plates at a density of 1000 cells per well. Three replicate wells were set up for each sample. Cell proliferation was examined daily for 5 d, consecutively, after cell seeding. On each day, 10 µl of the CCK-8 solution was added to each well of cells. After 1 h of incubation, the absorbance of each well was measured using a microplate reader, after which the results were statistically analyzed. The plate clone formation assay was conducted using 6-well plates (Corning Inc.). Briefly, the cells were seeded into the 6-well plates at a density of 300 cells per well and cultured for 10 d. Subsequently, the cells were fixed with 4% paraformaldehyde for 30 min, rinsed in running water and stained with crystal violet for 15 min. The cells were washed in slow running water and the number of clones was counted.

### Examination of apoptosis and cell cycle

The A549 and SPC-A1 lung adenocarcinoma cells were seeded into 6-well plates and transiently transfected with siRNA. At 48 h after transfection, the cells were collected. The cells were then washed once with phosphate-buffered saline (PBS), double-stained with FITC-Annexin V and propidium iodide (PI) for 15 min in the dark and analyzed using flow cytometry (FACScan®; BD Biosciences, San Jose, CA). The cell cycle was examined using a commercial kit (Multi Sciences (Lianke) Biotech Co., Ltd.) in accordance with the manufacturer’s instructions.

### Examination of cell migration and invasion

Cell migration assay: At 24 h after transfection, the cells were harvested and counted. Fifty thousand cells were added into the upper chambers of the transwell plate (Corning Inc; Pore size, 8 μm) and overlaid with 200 μl serum-free medium. One milliliter of serum-containing medium was added to the lower chambers. After 24 h of incubation, the cells were fixed with 4% paraformaldehyde for 30 min, washed 3 times with PBS (Gibco) and stained with 0.1% crystal violet for 15 min (Beyotime Biotechnology Corporation). After washing under slowly running water, the cells were imaged and counted under a microscope. Cell invasion assay: The upper chambers of the transwell plates were pre-coated with Matrigel (Sigma-Aldrich, USA). The remainder of the procedure was the same as for the migration assay.

### Tumor formation in nude mice

Nude mice (specific-pathogen-free (SPF) grade, 4 weeks of age) were obtained from the Experimental Animal Center of Guangdong Province. Each group contained 6 mice (half male and half female). The mice received subcutaneous inoculation of 5 × 10^6^ treated A549 cells. The data were collected 15 d after inoculation.

### Tail vein injection

A549 cells in which lncRNA CASC9.5 was stably knocked down and the blank control group were generated by transfection with small hairpin RNAs (shRNAs). The cells were collected and cell concentration was adjusted to 2 × 10^7^ cells/ml. Mice (4 weeks of age) were injected with 100 μl of cells via the tail vein. The data were collected at 7 w after the injection. The lung tissues were collected, and visible masses were examined and counted.

### Statistical analysis

SPSS v13.0 statistical software was used for data analysis. Measurement data were expressed as the mean ± standard deviation ($\overline{{\rm{x}}}\pm {\rm{s}}$), and a t-test was performed for between-group comparisons. One-way analysis of variance (ANOVA) was performed to compare the mean values among groups, and the least significant difference (LSD) t-test was performed for pairwise comparisons. A value of α = 0.05 was set as the significance level, and p < 0.05 was considered statistically significant (*p < 0.05; **p < 0.01; ***p < 0.001).
